# Fibrin-based clot firmness as assessed by rotational thromboelastometry is predictor of total amount of chest tube output in patients following elective cardiac surgery

**DOI:** 10.1186/1749-8090-8-S1-P84

**Published:** 2013-09-11

**Authors:** K Praprotnik, M Petricevic, B Gersak, B Biocina

**Affiliations:** 1 School of Medicine, University of Ljubljana, Ljubljana, Slovenia; 2 Department of cardiac surgery, KBC Zagreb, Zagreb, Croatia

## Background

Excessive bleeding after cardiopulmonary bypass (CPB) is risk factor for adverse outcomes after elective cardiac surgery (ECS). Differentiation between patients who bleed due to surgical issues and those whose excessive chest tube output (CTO) is due to hemostatic disorder remains challenging. Study aim was to evaluate prediction of excessive bleeding after ECS using fibrin-based clot firmness parameter of rotational thromboelastometry (FIBTEM test).

## Methods

We enrolled 148 patients (105 male and 43 female) undergoing ECS in a prospective observational study. Patients were found to be bleeders if their total amount of chest tube output exceeded 75th percentile of distribution. FIBTEM test was performed at three time points: preoperatively (T1), during CPB (T2), and after protamine administration (T3). Fibtem test values were correlated with amount of chest tube output and compared between patients with respect to excessive bleeding presence.

## Results

Total amount CTO value of 1505 ml presented 75th percentile of distribution, thus cut off value of bleeder category. Bleeders had longer duration of CPB (median 116 vs. 95 min, p=0.023) and lower value of the lowest body temperature during CPB (30 vs. 32 C°, p=0.012). With respect to excessive bleeding presence we observed significantly different FIBTEM test values between patients at T1 and T3 time point. Although FIBTEM test values in all three time points significantly correlated to CTO, the strongest correlations were observed at T3 (FIBTEM A30, Spearman r = -0.25, p=0.002). Conventional laboratory fibrinogen value, as expressed in g/l failed to correlate with CTO (r=-0.154, p=0.062).

## Conclusion

Our study showed that FIBTEM test is useful in predicting excessive bleeding after ECS. In order to prevent excessive postoperative CTO, hemostatic intervention with timely and targeted fibrinogen concentrate therapy according to FIBTEM test results should be considered.

